# Person-centred medicine in the care home setting: feasibility testing of a complex intervention

**DOI:** 10.1186/s12875-025-02925-8

**Published:** 2025-08-25

**Authors:** Line Due Christensen, Hilary L. Bekker, Flemming Bro, Anne Estrup Olesen, Jette Kolding Kristensen, Kirsten Høj

**Affiliations:** 1 Research Unit for General Practice, Aarhus, Denmark; 2 Leeds Institute of Health Sciences, University of Leeds, Leeds, UK; 3 Research Centre for Patient Involvement, Aarhus University Hospital, Aarhus, Denmark; 4 Department of Public Health, Aarhus University, Aarhus, Denmark; 5 Department of Clinical Pharmacology, Aalborg University Hospital, Aalborg, Denmark; 6 Department of Clinical Medicine, Aalborg University, Aalborg, Denmark; 7 Centre for General Practice, Aalborg University, Aalborg, Denmark; 8 Department of Clinical Pharmacology, Aarhus University Hospital, Aarhus, Denmark

**Keywords:** Patient-centred medicine, Medicines optimization, Residential care, Elderly care, Primary health care, Normalization process theory, Feasibility testing, Denmark

## Abstract

**Background:**

Person-centred medicine in older patients requires medication decisions to be aligned with individual preferences, needs, and values. However, involvement of care home residents and their relatives in such decisions remains limited due to professional preferences and perceived barriers. This study investigates the feasibility of a newly developed intervention aiming to facilitate person-centred medicine through resident and relative involvement and interprofessional communication support.

**Methods:**

The feasibility testing was conducted in two care homes from April to October 2022 in an urban Danish Municipality. The intervention consisted of two components: the PREparation of Patients for Active Involvement in medication Review for Care Home (PREPAIR-CH) and a medication communication template for healthcare professionals. A flexible three-stage workflow and a multifaceted implementation strategy facilitated implementation. Data was collected through observations and interviews with healthcare professionals (care home staff, GPs), residents, and relatives. Data analysis was guided by Normalization Process Theory.

**Results:**

Ten residents participated in the intervention (four in the presence of relatives) and were subsequently interviewed. Additionally, five interviews with healthcare professionals were conducted. The intervention purpose was deemed relevant by residents, relatives, and healthcare professionals and aligned with individual values. The implementation strategy followed the intended delivery. Flexibility, coordination, and collaboration within the local team were key to facilitating intervention implementation. Challenges included selection of residents, involvement of relatives, and management of competing priorities. The intervention offered a structure for involvement and provided valuable insights for healthcare professionals into the patient perspective, thereby fostering reflection and dialogue and enhancing the residents’ and relatives’ perceived involvement. The medication communication template was considered relevant by staff, whereas GPs found it unnecessary.

**Conclusions:**

The PREPAIR-CH was found acceptable and feasible by residents, relatives, and healthcare professionals, but care home staff and GPs disagreed on the relevance of the medication communication template. The findings suggest that the intervention may enhance resident and relative involvement to support person-centred medicine. Some uncertainties must be explored before a large-scale evaluation, including the applicability to different types of residents and how to support interprofessional communication about medicines, as the needs appear to differ between care home staff and GPs.

**Supplementary Information:**

The online version contains supplementary material available at 10.1186/s12875-025-02925-8.

## Background

Person-centred medicine is recommended in the care of older patients [1, 2]. It involves medication-related decisions guided by an individual’s preferences, needs, and values [3]. Medication-related decisions in older patients are highly sensitive to preferences, as treatment guidelines are often based on young, healthy patient populations that may not generalize to older patients [4–6]. Incorporating individual preferences and priorities into medical decision-making can improve treatment adherence, patient satisfaction, perceived well-being, and quality of life [7–14]. Yet, in the care home setting, involvement of residents and their relatives in medication-related decisions remains limited [15–19].

Well-known barriers to involving residents and relatives are awareness and attitudes towards involvement among health care professionals (HCPs) [20]. Research shows that HCPs may perceive care home residents as uncapable of or uninterested in being involved in their own care [21]. Furthermore, involving relatives is sometimes perceived as time-consuming, not helpful, and sometimes even problematic [22]. Research emphasizes that many residents and relatives wish to be involved [23–25], but residents often need support because they are unsure how to be involved or think that HCPs may not be receptive to their perspectives [26].

Recent reviews have assessed various tools for illuminating patient preferences in the context of multimorbidity and geriatric polypharmacy [27, 28]. So far, no ideal tool has been identified for use in a real-life clinical setting, although the need for simple and feasible methods has been stressed [27]. Implementing even a simple tool into a real-life care home setting involves changing the reasoning and actions of multiple stakeholders, such as residents, relatives, and HCPs. Additionally, it requires interprofessional coordination and communication.

Changing professional behaviour is a challenging task that warrants careful consideration of the specific context and existing care pathways. Interventions aiming to do so are often complex, e.g. contain multiple interacting components and/or require new behaviours of those delivering and receiving the intervention [29, 30]. A widely use framework for complex intervention research is the guidance developed by the Medical Research Council (MRC) for developing and evaluating complex interventions [30]. The MRC framework divides the research process into four phases: development (or identification) of the intervention, feasibility, evaluation, and implementation.

Based on the MRC framework, the overall aim of this study was to develop and test the feasibility of a complex intervention to support person-centred medicine in the care home setting through resident and relative involvement and interprofessional communication support. The development phase has been reported elsewhere [31]. This paper reports on the feasibility phase of the study, with a view to assessing the feasibility of the intervention and the implementation strategy from the viewpoint of HCPs and to assessing the feasibility of the intervention from the viewpoint of care home residents and their relatives.

## Methods

### Design

The study was designed as a non-randomized, small-scale feasibility study using qualitative evaluation methods. The overall approach adhered to the MRC framework for development and evaluation of complex interventions [30, 32]. The target population of the intervention was care home residents and their relatives, and the feasibility testing was performed in two care homes in Denmark from 1 April to 31 October 2022.

The study was conducted in close collaboration with Aarhus Municipality, Denmark, and was endorsed by the Danish Society for Patient Safety [33] and the municipal unit of the Organization of General Practitioners in the Central Denmark Region [34]. The CONSORT extension for feasibility studies [35] and the COREQ checklist for qualitative research (Supplementary material 1) guided the reporting of this study [36].

### Setting

According to Danish law, elderly and frail individuals who need all-day care are eligible for care home residency, and the municipalities are responsible for allocation [37]. In 2016, a new model with a designated general practitioner (GP) was introduced in Danish care homes [38]. In this model, one or several GPs are assigned to serve a specific care home while maintaining their private clinic. Most GPs in Denmark operate as independent contractors and are remunerated through the national health system. When residents move into a care home, they may retain their existing GP, but new residents are generally encouraged (but not obligated) to register with the designated GP at the care home. In Denmark, GPs are responsible for the majority of prescription medications [39] and for chronic care management [40].

The study took place in an urban municipality in the Central Denmark Region with a population of approximately 360,000 inhabitants. At the time of the study, 50 care homes in the municipality were affiliated with a designated GP.

### Intervention content and delivery

**Fig. 1:**
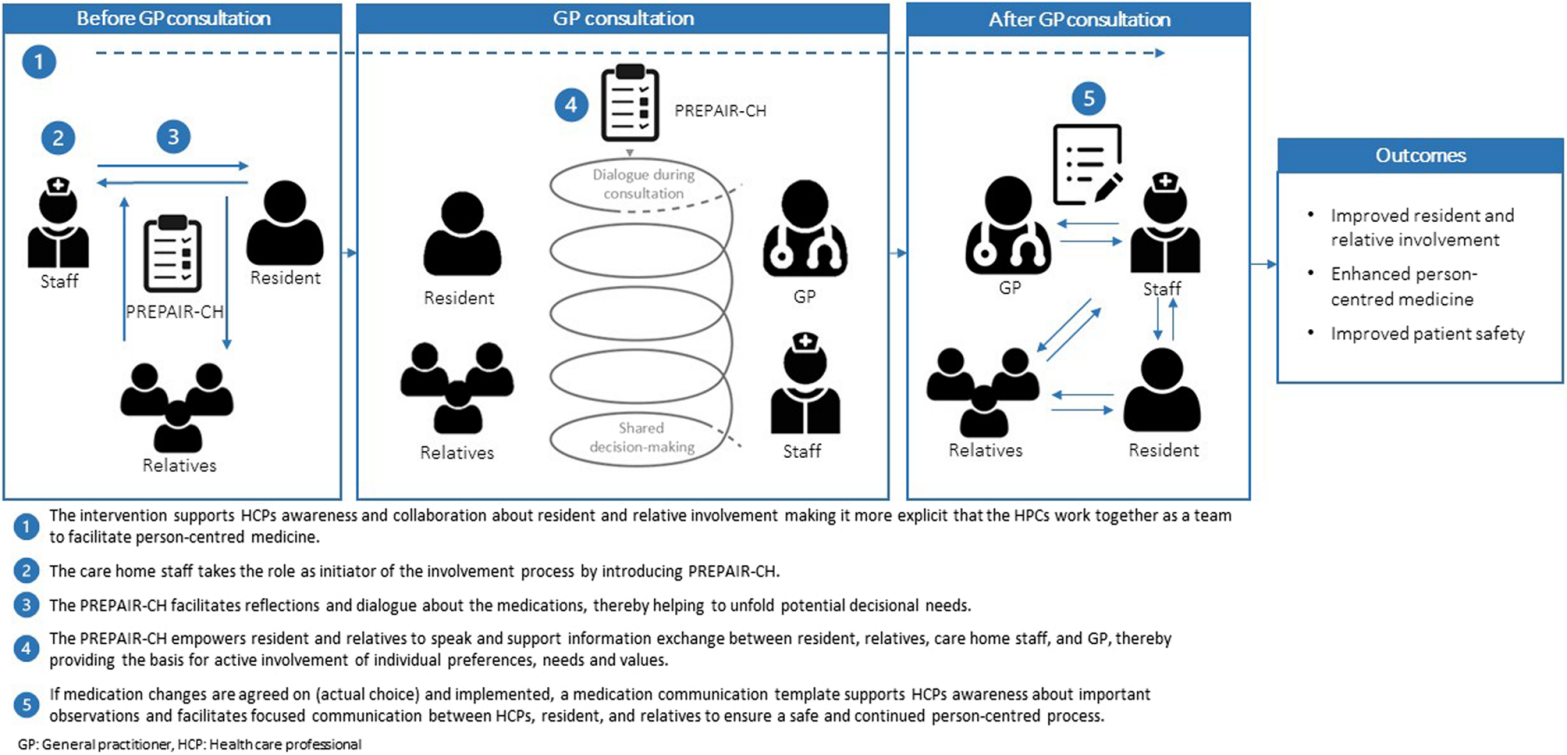
Linear logic model of the intervention, modified from Sandbæk et al. [42]

The intervention content is considered as *what* will be delivered, while the intervention delivery is considered to be *how* this is delivered [41]. In this study, the intervention content comprised two key components, which were delivered in a three-stage workflow (Fig. 1).

The two intervention components comprised the patient involvement tool “PREparation of Patients for Active Involvement in medication Review for Care Home” (PREPAIR-CH) and an interprofessional medication communication template. The PREPAIR-CH is a simple, five-item questionnaire to be completed by the care home resident with assistance from care home staff and/or relatives. The questionnaire uses a 3-point Likert scale for response options (Yes/No/Do not know) and includes an open text field (Supplementary material 2). The interprofessional medication communication template included fixed points related to medication changes and instructions for important observations (Supplementary material 3).

The intervention delivery included a three-stage workflow. First, the PREPAIR-CH was completed by the resident with support from the care home staff. This could be scheduled to align with the daily routine of the care home staff to ensure flexibility. Second, a GP consultation was conducted based on the PREPAIR-CH. During this consultation, the GP used the completed PREPAIR-CH as a starting point for discussing medication-related matters with the resident. The care home staff and relatives were encouraged to participate and facilitate the conversation if necessary and desired by the resident. Finally, follow-up on medication changes was mandated based on an interprofessional medication communication template, which was completed by the GP and forwarded to the care home staff as part of the overall care plan.

Figure 1 presents the linear logic model of the intervention, the proposed mechanisms of actions, and the expected outcomes. The PREPAIR study [42] and the Interprofessional Shared Decision Making model [43] were instrumental in shaping the linear logic model of the intervention. This provided valuable insights into key actors and their roles in supporting person-centred medicine in the complex care home setting.

### Implementation strategy

The implementation strategy comprised multiple components aimed at facilitating overall team engagement and knowledge, promoting adherence to the intervention, providing supportive leadership, and ensuring responsible implementation leaders (see components in Table 1).

**Table 1:** Components of the implementation strategy

Implementation strategy
Start-up meeting with the manager, selected care home staff, and the designated GP at the care home
Introduction/educational meeting with the entire care home staff group
Written information materials (e.g. project leaflet, intervention manual)
Appointed coordinators at each care home
Opportunity for contact with a researcher (telephone, e-mail)

Both HCPs (care home staff and GPs) received oral and written information about the project, intervention components, and delivery. No specific training was provided prior to the testing; the performance of the intervention was based on the HCPs’ existing clinical experience.

### Participants

The municipality selected two care homes to participate in the feasibility testing. The care homes were eligible if affiliated with a designated GP. The designated GPs affiliated to the participating care homes were then invited to participate. At each care home, the care home managers identified relevant care home staff to be allocated to the study. The care homes had 40 and 36 residential care units, respectively. To minimize the study-related burden on the care homes, a pragmatic sample size of ten residents was chosen. In line with the concept of information power [44], this sample size was deemed sufficient to address the study’s research foci.

At each care home, five residents were purposively sampled by care home staff and GPs in collaboration and invited to participate. Care home staff and GPs identified eligible residents based on their knowledge about the residents’ cognitive status and communication abilities. This was a pragmatic approach to ensuring the recruitment of residents that were able to participate meaningfully both in the intervention and the subsequent interview. Furthermore, it served to engage care home staff and GPs and reflect a real-life clinical setting where they are responsible for the interactions with residents and relatives. Inclusion criteria for residents included the ability to provide informed consent and to complete the PREPAIR-CH with support from the care home staff. A single exclusion criterion was severe cognitive impairment that could not be accommodated by support from care home staff or relatives.

To adapt the recruitment process to the everyday routines and relationships within the care home setting, all care home residents and participating relatives received oral study information from the care home staff, in addition to the provision of written study information (leaflets) drawn up by the researchers. None of the invited residents declined to participate. It was not possible for all residents to involve a relative. In total, ten care home residents, four relatives, five care home staff, and two GPs consented to participate in the feasibility testing, observation, and subsequent interviews. All participants provided written informed consent before participation. All the participating residents had varying degrees of cognitive impairment.

### Data collection

A multi-faceted approach was used to evaluate this feasibility study, including observations and semi-structured interviews to gain comprehensive insights into the feasibility of the intervention and the implementation strategy. Observations were performed in connection with delivery of the implementation strategy and during all GP consultations in the two care homes. During the GP consultations, the primary focus was on how the PREPAIR-CH was applied in practice as well as on naturalistic individual behaviours and interactions between residents, relatives, care home staff, and GPs. We used a complete observer approach, where the researcher observed without participation [45]. No formal observation protocol was used, but the observations and subsequent field notes were guided by our pre-defined research foci while still allowing for new insights. Descriptive and reflexive notes were made immediately after visits to the care homes to ensure accuracy and reduce the risk of inaccuracies due to delayed recall. Observations of GP consultations lasted between 15 and 30 min.

After the GP consultation, semi-structured interviews were conducted with residents and relatives to explore their experiences with the intervention. After completion of all intervention tests, semi-structured interviews were conducted with the HCPs to explore their experiences with the intervention and the implementation process. The interviews were audio-recorded and conducted at the care homes or (for GPs) in the clinics. The duration of the interviews with residents and relatives lasted between 10 and 30 min, depending on participant availability and engagement. Interviews with GPs and care home staff ranged from 30 to 45 min. The interview guides were based on Normalization Process Theory (NPT), which offers an explanatory framework for understanding the mechanisms of implementing interventions in healthcare settings [46] (supplementary material 4). NPT focuses on the work of individuals and groups needed to integrate an intervention into routine practice. It encompasses four fundamental components: coherence, cognitive participation, collective action, and reflexive monitoring. The interview guide for residents and relatives included questions from three existing scales that were planned to serve as outcome measures in a later evaluation phase [47–49] (Supplementary material 4). Observations and interviews were carried out by LDC.

### Data analysis

Systematic text condensation [50] with a rapid analysis approach [51] was used for the data analyses. Systematic text condensation provided a structured framework for identifying and synthesizing key themes. The rapid analysis was inspired by the approach described by Neal et al. [51], in which prespecified key research foci are identified directly from audio recordings, which reduces time-consuming verbatim transcriptions and line-by-line coding while still capturing essential information and allowing for new themes to emerge.

First, all field notes were read, and audio-recordings were listened through for total impression by LDC. Second, data units related to key research foci (intervention feasibility, implementation processes, and participant experiences) were identified, transcribed (for audio-recordings), and coded through an iterative process, in which the coding list was continuously refined. An inductive approach was used at this stage to allow for new insights to emerge. Third, codes were condensed into sub-themes which were subsequently categorized deductively under the overarching construct of the NPT. This process was undertaken by LDC and KH, and the results were discussed and agreed upon by all authors. Data analyses were conducted in NVIVO 1.7.1 software. Finally, selected citations from the interviews were translated to English using linguistic service and included to enhance transparency of results.

## Results

The implementation strategy was delivered as intended during the feasibility testing (Table 1). We conducted ten resident interviews (of which four included relatives) and five HCP interviews (Table 2). The key findings emerging from the analyses of observational and interview data were organized thematically according to the four NPT constructs: coherence, cognitive participation, collective action, and reflexive monitoring [46].

**Table 2:** Overview of participants

Care home	Interview no.	Pseudonym	Type of participant	Gender
CH1	1	RS1	Resident	Female
		R1	Relative to RS1 (daughter)	Female
	2	RS2	Resident	Female
		R2	Relative to RS2 (daughter)	Female
	3	RS3	Resident	Female
		R3	Relative to RS3 (daughter)	Female
	4	RS4	Resident	Female
	5	RS5	Resident	Female
	6	GP1	General practitioner	Male
	7	N1	Nurse	Female
CH2	8	RS6	Resident	Male
		R4	Relative to RS6 (wife)	Female
	9	RS7	Resident	Female
	10	RS8	Resident	Male
	11	RS9	Resident	Female
	12	RS10	Resident	Female
	13	GP2	General practitioner	Female
	14	N2	Nurse	Female
		N3	Nurse	Female
	15	SoHA 1	Social and health care assistant	Female
		SoHA 2	Social and health care assistant	Female

### Coherence

Coherence relates to the participants’ individual and collective understanding of the intervention. During the interviews, the residents and HCPs articulated their reflections on the intervention and their motivations to participate. Additionally, observations during implementation strategy delivery provided insights into the participants’ attitudes towards the intervention.

#### Alignment with individual values and motivations

The primary purpose of the intervention was to support person-centred medicine in the care home setting through resident and relative involvement and interprofessional communication support. This purpose was found to align with the values and viewpoints of both residents and HCPs.

The residents reported an unmet need for support to be involved in conversations about their medications. All ten residents experienced that decisions about their medication were usually mainly made by the GP and/or the care home staff. Many of the residents wished to be informed about any changes in the treatment. However, the residents did not always know how to be actively involved. A resident specified:

*“I would like to know the reason why it [blood pressure medication] is still necessary*,* but I don’t really know much about it after all” (RS4*,* CH1).*

The HCPs perceived the intervention as relevant and meaningful. A nurse explained:

*“It makes good sense that our residents are informed about their medication*,* are involved in it*,* and can tell their side of the story. And again*,* yes*,* we do also have several who are cognitively impaired*,* but then we have some relatives*,* and I think that it’s important that they are also heard” (N1*,* CH1).*

The HCPs also found it motivational to work with their procedures and workflows to improve patient care. A GP articulated her considerations this way:

*“[It is motivational to explore] whether there is something that could be done easier*,* smarter*,* more desirable for the patients that I deal with” (GP2*,* CH2).*

Moreover, the HCPs perceived the intervention as useful for enabling deprescription of unnecessary or inappropriate medication, which is an important aspect of good patient care. A SoHA explained:

*“For me*,* it was more this thing that there might be medications they could do without. Do they perhaps get something they don’t need? Because I think that often more [medicine] is just added” (SoHA1*,* CH2).*

In one care home, deprescribing among residents was already a priority prior to the intervention.

The observable behaviours and interactions during introduction meetings, which were conducted as part of the implementation strategy, also expressed positive attitudes among the care home management as well as the care home staff and GPs towards the project in agreement with the verbal expressions during interviews. Hence, the intervention was found to align well with both individual and organizational values.

### Cognitive participation

Cognitive participation covers how the participants engage in and plan the implementation of the intervention. During the interviews, the HCPs voiced different factors that facilitated and challenged this process.

#### Facilitators: flexibility, coordination, and collaboration

Both the designated GPs and the nurses valued the high degree of flexibility in the planning of the implementation of the intervention. This was emphasized as key to initiating the process. A nurse remarked:

“*Flexibility is the Alpha and Omega if you want it to lift off from the launching pad” (N2*,* CH2).*

In one care home, two nurses shared the task of coordinating, which was seen as vital. One of these nurses stressed the importance of having more than one coordinator:

*“That there is someone who can take over when the other one isn’t there. That*,* I believe*,* is almost a must” (N3*,* CH2).*

In this care home, residents eligible for inclusion were selected in collaboration between the two nurses and the designated GP. In the other care home, only one coordinator was appointed, as there was only one nurse employee at the care home. However, the nurse felt comfortable managing the intervention because of substantial support from the care home manager. Eligible residents were identified in a collaboration between the nurse and the care home manager and selected through discussion and agreement with the SoHAs who were thoroughly acquainted with the residents’ daily care needs and supported by the GP. Thus, flexibility, coordination, and collaboration within the local team were found to be key to facilitating intervention implementation.

#### Challenges: selecting the “right” patients, involving relatives, and managing competing priorities

Both care home staff and GPs found it challenging to determine which residents were relevant for inclusion in the feasibility testing, given that all residents had varying degrees of cognitive impairments. A GP expressed:

*“For me, it was relevant to find the right patients for this because many of the residents that I see are in a very, very bad state, and they would not be able to participate” (GP2, CH2).*

Some HCPs assumed that many residents were unable to participate due to cognitive impairments. This assumption was shared by one relative, who believed that she or the care home staff could speak on her mother’s behalf:

*“Mother forgets about it the moment that [care home staff name] turns her back and [she] would not be able to answer that again, and perhaps something else would come out [of it] next time. But I do know mum’s attitudes and such, and I also believe that [care home staff name] does”* (R2, CH1).

Observation showed that this relative was very satisfied with the GP conversation about the medicine based on the PREPAIR-CH, as she and her mother were explained about the various pills. In this example, the relative supported the resident in the conversation about the medication, which was helpful.

However, the nurses found it time-consuming to include the relatives, as they were often occupied and difficult to schedule appointments with. A nurse explained:

*“It takes some time; phoning them, writing them. They don’t pick up the phone. Can they [come to a meeting]? They need to find out and call back, and there’s a lot of coordinating” (N1, CH1).*

Additionally, competing priorities in the daily working routines limited the energy and time that the care home staff could allocate to intervention implementation. This was particularly evident during telephone conversations (part of the implementation support) with the researchers. Thus, including residents, particularly selecting the “right” patients, and involving relatives proved to be the main challenges for implementing the intervention, in addition to the challenge of managing competing priorities in routine care.

### Collective action

Collective action refers to the active work carried out in the intervention. Both observations and interviews provided insights into the intervention delivery, and some discrepancy was observed between intended and actual delivery.

#### Discrepancy between intended and delivered intervention

As intended, the PREPAIR-CH was completed by all participating residents with support from the care home staff and sometimes also from a close relative, e.g. spouse or daughter. In this process, some residents required assistance in verbalizing their expectations and preferences for their medication. In one care home, a nurse led this process. In the other care home, a SoHA (who was responsible for the daily care management) led the process.

Observations during the GP consultation showed that the PREPAIR-CH was actively used. Specifically, it was observed that the GPs had the PREPAIR-CH in their hands and used it as a basis for talking about the medication. This approach was also articulated by one of the GPs:

*“I hold it in my hand, but they are the ones with the answers after all. At some point, they have made up their mind about it, and you try to use their responses as the starting point, right?” (GP2, CH2).*

Additionally, it was observed in both care homes that a nurse participated in the GP consultation and played an active role by facilitating the conversation for the residents when necessary. In some cases, the nurse provided relevant information on the resident’s current and previous medication.

From the interviews, we discovered that none of the GPs used the interprofessional medication communication template in their communication with the care home staff after the consultation between GP and resident. Instead, the GPs relied on their usual modes of communication.

Overall, the observations and interviews showed converging findings suggesting that the intervention was feasibly delivered as intended in the first two stages of the workflow. However, a discrepancy between intended and delivered intervention was also present, as the interprofessional medication communication template was not used as intended in the last stage during follow-up.

Reflexive monitoring.

Reflexive monitoring concerns the participants’ perspectives on the intervention after having carried it out. During the interviews, the HCPs and the residents and relatives voiced various perspectives on the intervention.

#### HCP perspectives: new insights and structure

The care home staff and GPs shared a common experience when using the PREPAIR-CH during their dialogue with the resident; it illuminated the residents’ expectations and preferences for their medication, thereby revealing insights that might otherwise have remained unknown to the HCPs. A nurse expressed this newfound insight:

*“They came up with some things that made me think ’there are some things after all that we perhaps do not hear so much about’*,* but when they are asked directly*,* then something appears. […] That has given something*,* I believe*” (N1, CH2).

Additionally, one of the GPs highlighted the issue of expectations:

*“One of my patients had an expectation to something [medication review]*,* and I never thought that she would actually convey this expectation […] It was actually quite nice to learn more about it*” (GP1, CH1).

Both GPs and care home staff noted that the intervention provided a structured approach to facilitate resident and relative involvement. A GP stated:

*“I gain insight into the other side [of the matter], where someone, sitting in the chair, must take all [the medications] that I am prescribing, and this has become a little more systematized” (GP1, CH1).*

The GP further envisioned that the PREPAIR-CH could be a natural component of annual chronic care consultations in the future:

*“So*,* it becomes part of an actual set-up*,* where relatives are invited*,* and the elderly resident is in focus*,* and a proposal will be presented*,* and we can discuss different things*,* where it will form part” (GP1*,* CH1).*

The nurses also believed that a more systematic approach to involving residents and their relatives might reduce unnecessary worries and contacts from the relatives. A nurse said:

*“Then perhaps you can also avoid relatives becoming insecure and calling”* (N2, CH2).

For the HCPs, the intervention yielded new insights into the perspectives of residents and relatives, and it delivered a structure to support involvement in medication-related decisions.

#### Conflicting views on interprofessional communication about medication

Contradictory views were observed between the care home staff and the GPs regarding the interprofessional medication communication template. According to the care home staff, this intervention component could facilitate the delivery of important information that would be helpful for the care home staff. A nurse explained:

*“I would say that you are actually on to something here that I could miss because we are not always informed about the observations that we should be attentive to. Then we can go in and figure it out ourselves*,* but it would be nice if it was included in his [the GP’s] correspondences”* (N1, CH1).

In contrast, the two GPs found their usual communication methods with the care home staff to be adequate and saw no need for introducing the interprofessional medication communication template. One of the GPs stated:

*“There will also be some dialogue when medication is initiated. And if there is something of significance that should be kept an eye on for some medication*,* then information will also be given about it. But generally*,* information is not given about all adverse effects” (GP1*,* CH1).*

These differing viewpoints revealed different needs and expectations between care home staff and GPs with respect to communication about the residents´ medication.

#### Resident and relative perspective: reflection and person-centred dialogue

Some residents and relatives expressed that using the PREPAIR-CH facilitated reflection on the medication. One resident felt inspired by the tool to write down questions about her medication:

*“In the future, I will just – if I remember something – write it down… you often think, ’What about this? What should I do here? Whom should I ask about it?’ And then this questionnaire comes, and you can put it all in here”* (RS7, CH2).

Some relatives found that the PREPAIR-CH facilitated an important dialogue with HCPs about the medication. One relative described it as follows:

*“We have… like… talked about the problem, and then we have found a solution together” (R1, CH1).*

Another relative commented:

*“It’s nice enough to enter the scene and know what’s going on. Also because we can talk to my mother about it*” (R3, CH1).

Both observations and interviews with residents and relatives indicated that the PREPAIR-CH effectively promoted a patient-centred approach during conversations with the HCPs about medication. Residents and relatives felt actively involved, with some expressing that the GPs were more attentive during the consultation. A resident expressed:

*“He has obviously taken into consideration all the things that I asked about and tries to look into the possibility for me to get rid of some pills”* (*RS4*,* CH1).*

Overall, the intervention, particularly the PREPAIR-CH, was found to encourage reflection, preparation, and person-centred dialogue, which, in turn, enhanced the residents’ and relatives’ feeling of being involved and heard.

## Discussion

### Main findings

The feasibility testing of this complex intervention and implementation strategy was analyzed through the lens of NPT. Themes related to coherence showed alignment between the intervention purpose and the individual values and motivations of the participants. Themes related to cognitive participation included facilitators (flexibility, coordination, and local collaboration) and challenges (selecting the “right” residents, involving relatives, and managing competing priorities) to implementation. Collective action themes showed some discrepancy between actual and intended intervention delivery, as the interprofessional medication communication template was omitted by the GPs. Reflexive monitoring themes revealed conflicting viewpoints between care home staff and GPs on this intervention component as they had different interprofessional communication needs and expectations. Nevertheless, the intervention fostered reflection and person-centred dialogue, thereby enhancing residents’ and relatives’ sense of involvement. Furthermore, it provided new insights and a structure for the HCPs to support patient involvement and person-centred medicine.

### Comparison with existing literature

Research shows that care home residents often experience that HCPs make the decisions about their medications [25, 52, 53]. Studies further indicate that residents generally wish to be involved [25, 54, 55], but that they often find this difficult [25, 52]. These viewpoints were also articulated by the residents in our study. In our study, residents, relatives, and HCPs all considered resident and relative involvement to be highly relevant and meaningful. Still, an important barrier to getting started with the intervention was selecting the “right” patients as perceived by the HCPs. Care home residents are a very heterogeneous group with different health conditions and complex needs. Hence, recruitment of residents who are able to consent and participate can be challenging, and this has been reported to be the most difficult part of research in care home facilities [56, 57]. A contributing factor may also be the observed preconception among HCPs that many residents are incapable of participating, which has been highlighted in previous research [18, 22, 56].

A way of supporting cognitively impaired residents in being involved is through the involvement of relatives who know the resident and can speak on their behalf [57, 58]. In our study, the relatives found it satisfying to be involved as they were more informed themselves, able to support the resident, and empowered to speak on the resident’s behalf in case of cognitive impairment. However, recruiting relatives was perceived as challenging by the HCPs, mostly due to practicalities and staff time constraints. Other studies have shown that both designated GPs and care home staff may have some reservations pertaining to involvement of relatives in medication-related decisions, such as concerns about the time needed to involve relatives and that the personal views of the relatives might not always be helpful [22, 56]. Although these concerns were not articulated by the HCPs in our study, such concerns could be potential barriers to resident involvement that are important to consider.

In our study, the identified facilitators for implementation were flexibility, coordination, and local collaboration, which were supported by the implementation strategy. These facilitators contributed to integrating the intervention into daily routine care, and they ensured balanced fidelity to the intended intervention and adaptation to local contextual needs. The intervention and implementation strategy tested in our study align with theories about mechanisms to successfully implement patient decision aids in routine healthcare settings [59]. According to these theories, key facilitators of successful implementation in healthcare contexts include organisational priority of an intervention, co-production of implementation strategies with end-users, and engagement of the entire healthcare team while providing information about purpose and intended use of the intervention [59].

The intervention was found to provide a structure that facilitated resident and relative involvement by increasing the awareness among HCPs and disrupting the habits of usual care. Using the PREPAIR-CH empowered the residents to speak and brought new insights into the patient perspectives for the HCPs. From the viewpoint of residents and relatives, the PREPAIR-CH facilitated reflection and dialogue about the medication, which enhanced their feeling of being heard and involved. These results were consistent with the findings during the intervention development phase and supported our programme theory [31]. A contrasting finding was the conflicting views on the interprofessional medication communication template, as this was supported as an intervention component by both GPs and care home staff in the development study [31]. This emphasizes that different healthcare professionals in different organisations may have different views on and needs for interprofessional collaboration, which may affect intervention implementation and local adaption needs at different sites.

### Implications

The findings of this feasibility study suggest that the intervention is acceptable and feasible and that it may indeed improve resident and relative involvement and support person-centred medicine. The intervention was performed by the regular care home staff and GPs at the care homes with no additional resources added. This suggests that the intervention is realistic and sustainable in a real-life setting. However, key uncertainties remain and need to be addressed. These include exploring whether the intervention applies to all residents or only a selected group of residents with mild to moderate cognitive impairment and, in this process, gain a deeper understanding of the barriers and facilitators to reaching the target population. As proposed by the HCPs in this study, a way to explore this is to use the PREPAIR-CH systematically for all residents e.g. as a natural component of the annual GP status consultations in the care homes. Other uncertainties comprise identifying the most operational workflow of the intervention and understanding the different needs and expectations for communication on medicines in care home staff and GPs following the GP consultation. These uncertainties need to be explored in future studies of the intervention.

Ultimately, a large-scale evaluation is needed to determine whether the intervention can improve short-term outcomes, such as resident and relative involvement, and whether this may improve outcomes of importance to patients and society, such as improved quality of life and fewer emergency department visits and hospitalizations. Additionally, an upscaling of the intervention may reveal unintended consequences or harms that were not identified during the development phase and small-scale feasibility testing. For instance, involvement may burden the relatives by inducing worries or feelings of guilt [55, 60].

### Strengths and limitations

A strength of this study was the use of the NPT framework to guide the data collection and analysis. The NPT framework helped understand the feasibility of the intervention, and how the intervention was implemented in a care home setting. NPT is increasingly used for qualitative analyses of implementation activities across a diverse range of health care contexts [61, 62]. Furthermore, it has been suggested as a prospective tool to heighten the awareness of facilitators and barriers to successful implementation [62, 63]. The combined use of observations and interviews strengthened the credibility and the validity of the research findings. While the interviews uncovered the participants’ subjective experiences and perspectives, the observations captured actual behaviours and interactions in their natural settings, thereby providing important contextual insights which were found to be in agreement with the participant narrative. The participation of two care homes with no prior knowledge of the intervention before the feasibility testing further strengthened the validity and generalizability of our findings on acceptability and feasibility in the real-world care home setting.

A key limitation of this study was that the residents who agreed to participate were likely to have a better cognitive functioning than the average care home resident. Furthermore, the involvement of care home staff and GPs in selecting the residents may have contributed to the inclusion of residents with fewer observable challenges, as HCPs might unintentionally exclude residents perceived as highly vulnerable or unable to communicate about their medication. Consequently, important insights into the acceptability and feasibility of the intervention in the most vulnerable residents may not have been captured, potentially impacting the generalizability of the findings. Nonetheless, we successfully recruited and involved care home residents with mild to moderate cognitive impairment, all of whom required varying levels of support to participate. This patient group is often underrepresented in research, and their perspectives may reflect, to some extent, those of residents with more severe cognitive impairment who are unable to participate in interview studies. Another limitation concerning generalizability was the gender imbalance, with a predominance of female residents and relatives. This reflects the demographic composition of the care home population, where women are overrepresented due to greater longevity. Future studies in care homes should consider strategies to achieve a more balanced gender representation, where feasible, to ensure that a diversity of perspectives is adequately captured. Another limitation was the small number of included residents and relatives. Nevertheless, as their views were rather similar, their experiences are likely to be shared by other care home residents and relatives. Similarly, the viewpoints of HCPs were shaped by a small number of motivated individuals and may not generalize to other HCPs in care homes. Additionally, the researchers’ preconceptions might have impacted the findings. However, the interpretation of the results was carried out within a cross-disciplinary research group, which facilitated in-depth analyses and increased the awareness of any preconceptions.

## Conclusions

In this study, we conducted a feasibility test of a complex intervention aiming to support person-centred medicine in the care home setting through resident and relative involvement and interprofessional communication support. The main part of the intervention, including the PREPAIR-CH, was found acceptable and feasible for residents, relatives, and HCPs. In contrast, care home staff and GPs disagreed on the relevance of the interprofessional medication communication template. Overall, our results suggest that the intervention is likely to enhance resident and relative involvement, thereby supporting person-centred medicine in the care home setting. However, uncertainties remain regarding the applicability of the intervention to all residents and regarding our understanding of the different needs for and expectations to interprofessional communication about medicines, which may have important implications for local intervention implementation. These aspects warrant further investigation before a large-scale evaluation.

### Data availability

Data from the interviews (audio recordings and transcriptions) and observation (field notes) are saved on a secure server at the Research Unit for General Practice, Aarhus, Denmark, and are only available to the research team. Data can be made available on reasonable request by contacting the corresponding author.

## Supplementary Information

Supplementary Material 1.

Supplementary Material 2.

Supplementary Material 3.

Supplementary Material 4.

## Data Availability

The data generated during the current study are not publicly available due to the sensitive nature of the data and the personal information provided by participants. The data and other study materials are available from the corresponding author on reasonable request.

## Abbreviations

GDPR: General Data Protection Regulation
GP: General practitioner
HCP: Health care professional
MRC: Medical Research Council
NPT: Normalisation Process Theory
PREPAIR: PREparing Patients for Active Involvement in medication Review
PREPAIR-CH: PREPAIR care home
SoHA: Social and health care assistants
