# Supplementary material for: Person-centred medicine in the care home setting: feasibility testing of a complex intervention
Source: BMC Prim Care. 2025 Aug 25;26:265. doi: 10.1186/s12875-025-02925-8 (PMC12376713; doi:10.1186/s12875-025-02925-8)
Supplement: Supplementary file 2 — Supplementary Material 2. [file 12875_2025_2925_MOESM2_ESM.pdf]

Supplementary material 2

How do you feel about your medication?

You will soon consult your doctor to discuss your medication. This form may help you and your doctor decide whether your medication should be changed.

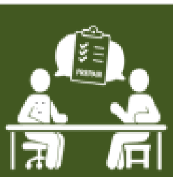

The questions concern your experience with the medication.

Do you experience adverse drug reactions that bother you significantly?	Yes	No	Do not know
Do you sometimes think that you get too much medication?	Yes	No	Do not know
Would you take less medication if your doctor said that it was possible?	Yes	No	Do not know
Are you overall satisfied with your current medication?	Yes	No	Do not know
Is there something about your medication that you or your relatives would like to discuss with the doctor?	Yes	No	

If yes, please elaborate: ______________________________

____

____

The questionnaire can be downloaded at www.feap.au.dk/PREPAIR.
Please select the **green** button with the questionnaire for residential care homes.

AARHUS UNIVERSITY

RESEARCH UNIT FOR GENERAL PRACTICE Aarhus
