# Supplementary material for: Person-centred medicine in the care home setting: feasibility testing of a complex intervention
Source: BMC Prim Care. 2025 Aug 25;26:265. doi: 10.1186/s12875-025-02925-8 (PMC12376713; doi:10.1186/s12875-025-02925-8)
Supplement: Supplementary file 3 — Supplementary Material 3. [file 12875_2025_2925_MOESM3_ESM.pdf]

Supplementary material 3

Box 1. Medication communication template

Plan: Information on the plan and agreements concerning medication changes

Observation: Information to the staff on important observations, including:

- Symptoms: e.g. withdrawal or relapse symptoms when deprescribing medication or potential adverse drug reactions if new medication is prescribed.
- Monitoring: e.g. weight, blood pressure, including when and how often.

Follow-up: Agreements concerning follow-up
