# Supplementary material for: Person-centred medicine in the care home setting: feasibility testing of a complex intervention
Source: BMC Prim Care. 2025 Aug 25;26:265. doi: 10.1186/s12875-025-02925-8 (PMC12376713; doi:10.1186/s12875-025-02925-8)
Supplement: Supplementary file 4 — Supplementary Material 4. [file 12875_2025_2925_MOESM4_ESM.pdf]

Supplementary material 4

Interview Guide for Semi-Structured Individual Interviews (GPs)

THEME:	Questions
**Background information**	**Canyoutell me a little about yourself?**<br>- Age<br>- Profession<br>- Facts about your practice (size, etc.)
**Engagement**	**What were your thoughts about the intervention when you were first introduced to it?**<br><br>**What motivated you to start using the intervention?**<br><br>**Was it clear to you how the intervention would benefit the patients?**<br><br>**What significance does it have that the Aarhus Municipality supports the intervention?**
**Uptake**	**Can you describe how your practice receives new interventions?**<br>- How does your staff respond to new interventions?<br><br>**Can you tell us about the introduction to the intervention?**<br>- Did you feel prepared to try out the intervention?<br>- If yes/no, in what ways? What was good or missing?
**The intervention**	**How have you/the care home specifically worked with the intervention?**<br>- How did you get started?<br>- Have you involved other staff in the trial?<br>- Have you created a plan/procedure for the work (in collaboration with…)?<br>- Have you used the accompanying manual or been inspired by it?<br>- Did you need another coordinator/responsible person for the trial?<br>- Was it important for you to participate in this intervention? Why?<br>- Did the intervention fit into the daily routines at the care home (as the care home GP)?<br>- How was the intervention incorporated into daily life?<br>- How does this intervention differ from your usual way of working?

**Patients Experiences**	**How did the patients receive the intervention?**<br>- What did the patients/relatives think about participating in the intervention?<br>- Can you tell us about some feedback you received from the patients/relatives who participated?<br>**How did you experience the conversation with the patients about medication?**<br>- During the completion of the PREPAIR-CH?<br>- During the GP's consultation?<br>- Was the conversation different from what you expected?<br>- What do you think about this way of involving patients and relatives?
**Maintenance**	**What has worked well with the intervention?**<br>- What are the advantages of the intervention? And for whom?<br>- What results do you believe the intervention yields?<br>**What has been most challenging?**<br>- What are the disadvantages of the intervention?<br>**Could the intervention become part of the usual procedure?**<br>- Are there the necessary resources?<br>- What adjustments (improvements) should we make for the intervention to work better at your care home?

Interview Guide for Semi-Structured Individual Interviews at Care Homes (Care home Staff)

THEME:	Questions
**Background information**	**Canyoutell me a little about yourself?**<br>- Age<br>- Employment at the care home<br>- Profession<br>- Facts about the care home (size, etc.)
**Engagement**	**What were your thoughts about the intervention when you were first introduced to it?**<br><br>**What motivated you to start using the intervention?**<br><br>**Was it clear to you how the intervention would benefit the residents?**<br><br>**What significance does it have that the Aarhus Municipality supports the intervention?**
**Uptake**	**Can you describe how your care home receives new interventions?**<br>- How does your staff respond to new interventions?<br>- Do you generally experience good support from the management?<br><br>**Can you tell us about the introduction to the intervention?**<br>- Did you feel adequately prepared to try out the intervention?<br>- If yes/no, in what ways? What was good or missing?
**The intervention**	**How have you/the care home specifically worked with the intervention?**<br>- How did you get started?<br>- Have you involved other staff in the trial?<br>- Have you created a plan/procedure for the work (in collaboration with…)?<br>- Have you used the accompanying manual or been inspired by it?<br>- Did you need another coordinator/responsible person for the trial?<br>- Was it important for the care home/you to participate in this intervention? Why?<br>- Did the intervention fit into the daily routines at the care home?<br>- How was the intervention incorporated into daily life?<br>- How does this intervention differ from your usual way of working?

**Residents Experiences**	**How did the residents receive the intervention (the PREPAIR-CH)?**<br>What did the residents/relatives think about participating in the intervention?<br>Can you tell us about some feedback you received from the residents/relatives who participated?<br><br>**How did you experience the conversation with the resident about medication?**<br>- During the completion of the PREPAIR_CH?<br>- During the GP's consultation?<br>- Was the conversation different from what you expected?<br>- What do you think about this way of involving residents and relatives?
**Maintenance**	**What has worked well with the intervention?**<br>- What are the advantages of the intervention? And for whom?<br>- What results do you believe the intervention yields?<br>**What has been most challenging?**<br>- What are the disadvantages of the intervention?<br>**Could the intervention become part of the usual procedure?**<br>- Are there the necessary resources?<br>- What adjustments (improvements) should we make for the intervention to work better at your care home?

Interview Guide for Semi-Structured Interviews (Care home Residents/ Relatives)

	Care home residents	Relatives
**THEME**	1) How long have you lived at this care	
**Background**	home?<br>2) When did you move in?<br>3) How would you describe your current health status?	1) What is your relationship to X [the resident]?<br>2) Do you live close to the care home? Do you visit often?<br>3) May I ask how old you are? Possibly, what do you do on a daily basis? (If you are retired: What was your occupation when you were working?)
**Involvement preference**	The following questions I would like to ask concern you and your medication.<br>The first question I would like to ask is:<br>1) As things are now, who decides which medication you should take?<br>a. Yourself<br>b. The GP(s)<br>c. Relatives<br>d. Care home staff<br>e. Others<br>2) How do you prefer decisions to be made about your medication?<br>a. Do you prefer to make the final decision regarding which treatment you should receive?<br>b. Do you prefer that you and your doctor share the responsibility for deciding which treatment is best for you?<br>c. Do you prefer to completely delegate decisions about your treatment to the GP?	1) As things are now, who decides which medication your relative should take?<br>a. Yourself<br>b. The GP(s)<br>c. Relatives<br>d. Care home staff<br>e. Others<br>2) How do you prefer decisions to be made about your relative's medication?<br>3) Can you describe how your relative is involved with the medication?<br>a. To what extent?<br>b. When?<br>c. What are your preferences?<br>4) (Alternatively, ask the same question to the relative, or allow them to participate in the discussion about involvement preference.)
**Before GP consultation**		
**PREPAIR Tool**	You have already completed the PREPAIR Tool…<br>1) How was your experience with completing the PREPAIR tool?<br>2) How did you complete the PREPAIR tool?<br>a. Alone<br>b. Together with a relative<br>c. Together with the care home staff<br>d. Together with the GP<br>3) Did the PREPAIR tool help you to reflect on your medication?<br>a. How?	1) How was your experience with completing the questionnaire?<br>2) Did the questionnaire help you to reflect on your relative's medication?<br>a. How?<br>3) Did the questionnaire help you to consider any questions you would like to ask the GP?<br>a. How?<br>4) Did the questionnaire prepare you to talk with the GP about what matters most to you regarding your relative's medication?<br>a. How?

	4) Did the PREPAIR tool help you to consider any questions you would like to ask the GP?<br>a. How?<br>5) Did the questionnaire prepare you to talk with your doctor about what matters most to you regarding your medication?<br>a. How?<br>*Additional questions if the questionnaire was completed together with the care home staff:*<br>1) Did the care home staff ask you how you feel about your medication?<br>Did the care home staff ask whether you had any questions or concerns regarding your medication that you would like to discuss with the GP?	
**<u>After</u> GP consultation**		
**Experienced involvement in medication-related decisions during the GP consultation**	When you think about the conversation you have just had with your GP...<br>5) How did you experience your conversation about your medication?<br><br>Follow-up questions:<br>a. Did the GP ask about your own experiences with your medication?<br>b. Were you able to discuss any questions or concerns you might have had about your medication with the GP?<br>c. Were you involved in the decision-making process when decisions were made regarding your medication?<br><br>(Optional supplementary questions:)<br>Did the GP ask what matters most to you in relation to your medication?<br>Were your priorities taken into account in the decisions about your medication?	1) Can you describe your experience of the conversation with the GP regarding how the medication was discussed?<br>a. Did the GP ask about your experiences with your relative's medication?<br>b. Were you able to discuss any questions or concerns you might have had about your relative's medication with the GP?<br>c. Were you involved in the decision-making process when decisions were made regarding your relative's medication?<br><br>2) (Optional supplementary question:)<br>Were you involved in decisions about your relative's medication to the extent that you would have liked?
**At last…**	1) How has it been to participate in the project?	1) How did you experience being involved in the project?<br>2) How did you experience X's [the resident] involvement in the project?
